# Adaptive Laboratory Evolution of *Cupriavidus necator* H16 for Carbon Co-Utilization with Glycerol

**DOI:** 10.3390/ijms20225737

**Published:** 2019-11-15

**Authors:** Miriam González-Villanueva, Hemanshi Galaiya, Paul Staniland, Jessica Staniland, Ian Savill, Tuck Seng Wong, Kang Lan Tee

**Affiliations:** 1 Department of Chemical & Biological Engineering and Advanced Biomanufacturing Centre, University of Sheffield, Sir Robert Hadfield Building, Mappin Street, Sheffield S1 3JD, UK; miriam.glez.villanueva@hotmail.com (M.G.-V.); hemanshigalaiya@gmail.com (H.G.); 2 Croda Europe Ltd., Oak Road, Clough Road, Hull HU6 7PH, UK; paul.staniland@croda.com (P.S.); jessica.staniland@croda.com (J.S.); ian.savill@croda.com (I.S.); 3 National Center for Genetic Engineering and Biotechnology, 113 Thailand Science Park, Phahonyothin Road, Khlong Luang, Pathum Thani 12120, Thailand

**Keywords:** *Cupriavidus necator* H16, *Ralstonia eutropha* H16, adaptive evolution, carbon co-utilization, glycerol, biodiesel, fat splitting, biopolymer

## Abstract

*Cupriavidus necator* H16 is a non-pathogenic Gram-negative betaproteobacterium that can utilize a broad range of renewable heterotrophic resources to produce chemicals ranging from polyhydroxybutyrate (biopolymer) to alcohols, alkanes, and alkenes. However, *C. necator* H16 utilizes carbon sources to different efficiency, for example its growth in glycerol is 11.4 times slower than a favorable substrate like gluconate. This work used adaptive laboratory evolution to enhance the glycerol assimilation in *C. necator* H16 and identified a variant (v6C6) that can co-utilize gluconate and glycerol. The v6C6 variant has a specific growth rate in glycerol 9.5 times faster than the wild-type strain and grows faster in mixed gluconate–glycerol carbon sources compared to gluconate alone. It also accumulated more PHB when cultivated in glycerol medium compared to gluconate medium while the inverse is true for the wild-type strain. Through genome sequencing and expression studies, glycerol kinase was identified as the key enzyme for its improved glycerol utilization. The superior performance of v6C6 in assimilating pure glycerol was extended to crude glycerol (sweetwater) from an industrial fat splitting process. These results highlight the robustness of adaptive laboratory evolution for strain engineering and the versatility and potential of *C. necator* H16 for industrial waste glycerol valorization.

## 1. Introduction

### 1.1. Glycerol in Industrial Biotechnology

Glycerol (propane-1,2,3-triol) is a constituent of triglycerides, typically produced from fat splitting or transesterification during soap or biodiesel production. Diverse applications of glycerol in cosmetics, toiletries, pharmaceuticals, food, and beverages have maintained a constant demand for this commodity chemical. Uncharacteristically, the supply of glycerol is currently dictated not by its own demand, but rather by the demand of biodiesel. A typical biodiesel plant generates 10% (w/w) of glycerol by-product per unit of biodiesel produced [1]. The increasing global demand and production of biodiesel have created a surplus of crude glycerol, thereby driving the glycerol price down. While there is little incentive to purify this crude glycerol given its low price and high water content, disposal of the crude glycerol presents both environmental and economic challenges due to the impurities present. This oversupply of crude glycerol has spurred the exploration of glycerol utilization to increase the value chain of biodiesel production. Potential applications include fermentation to high-value chemicals, digestion to produce biogas, and pyrolysis and liquefaction for bio-oil production [2,3].

Microbial fermentation of glycerol into high-value chemicals such as 1,3-propanediol, alcohols, short-chain organic acids, polyhydroxyalkanoates, and hydrogen is a very promising application of crude glycerol [3]. The productivity of various microorganisms using crude glycerol as feedstock has been comprehensively reviewed [4]. While natural pathways for glycerol metabolism exist in many microorganisms, some are unsuitable for industrial applications due to their potential pathogenicity, like *Enterococcus faecalis* [5], *Listeria monocytogenes* [6], and *Mycoplasma* spp. [7]. Other requirements, such as the availability of genetic engineering tools to introduce and tune metabolic pathways, further reduce the number of useful microorganisms for industrial glycerol biotransformation.

### 1.2. *Cupriavidus necator* H16 Can Produce Polyhydroxyalkanoate from Glycerol

A promising candidate for glycerol biotransformation is the non-pathogenic Gram-negative bacterium *Cupriavidus necator* H16. Best known for its natural ability to accumulate polyhydroxybutyrate (PHB) [8], recent literature has evidenced a rapid expansion of *C. necator* H16 applications [9] and the development of its genetic engineering tools [10]. While *C. necator* H16 can naturally metabolize glycerol, carbohydrates, lignin derivatives, organic acids, and even carbon dioxide [11], its growth in glycerol is slow compared to favorable carbon sources like gluconate and fructose. Herein, we applied adaptive evolution to improve the glycerol assimilation in *C. necator* H16 leading to a variant that enables co-utilization of glycerol and gluconate. The isolated variant has a specific growth rate in glycerol that is 9.5 times faster than the wild-type. It utilizes gluconate and glycerol simultaneously, while the wild-type uses them sequentially. It also accumulates more PHB in glycerol medium than in gluconate medium, while the inverse is true for the wild-type strain. Genome sequencing and expression studies identified glycerol kinase as the key enzyme for improved glycerol utilization. Enhanced glycerol assimilation in the v6C6 variant was also extended to crude glycerol (sweetwater) from an industrial fat splitting process.

## 2. Results and Discussion

### 2.1. Glycerol Metabolism in *C. necator* H16 Wild-Type

**Figure 1:**
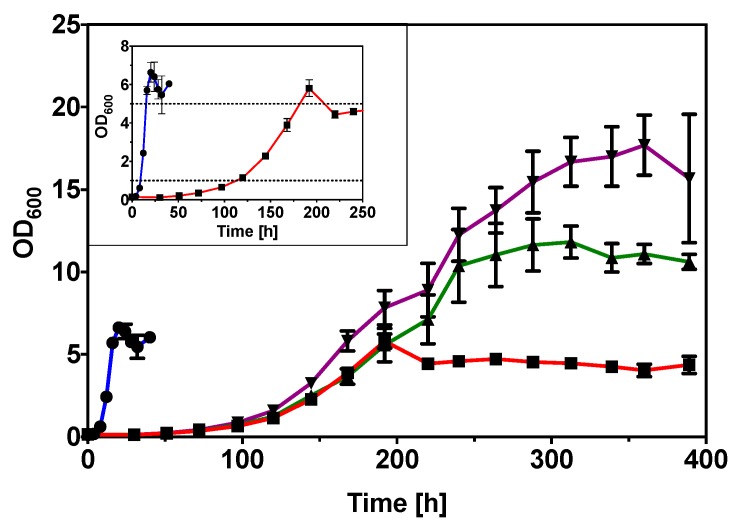
Growth curves of *C. necator* H16 wild-type in gluconate and laboratory-grade glycerol; 0.59% (*w*/*v*) sodium gluconate (blue line; circle), 0.50% (*w*/*v*) glycerol (red line; square), 1.00% (*w*/*v*) glycerol (green line; triangle), and 2.00% (*w*/*v*) glycerol (purple line; inverted triangle). The inset shows the magnified growth curve of *C. necator* H16 wild-type in 0.59% (*w/v*) sodium gluconate (blue line; circle) and 0.50% (*w/v*) glycerol (red line; square), with the dotted lines indicating OD_600_ of 1.0 and 5.0.

The growth of *C. necator* H16 at different glycerol concentrations (0.50%, 1.00%, and 2.00% (*w*/*v*)) were determined and compared to sodium gluconate (0.59% (*w*/*v*)), a preferred carbon source. The concentrations of 0.50% (*w*/*v*) glycerol and 0.59% (*w*/*v*) sodium gluconate were deliberately chosen to maintain the same carbon molarity (0.16 M) and *C. necator* H16 showed similar final optical densities (OD_600_) of 6.0 to 7.0 in both carbon sources at this carbon concentration. As shown in Figure 1, the growth in glycerol was slower than that in sodium gluconate. Growth in glycerol was characterized by a long lag phase of 110 h to reach the OD_600_ of 1.0, and a further 70 h before it achieved OD_600_ of 5.0 (late-exponential phase). This slow growth in glycerol required 180 h (i.e., 7.5 days) and is 12-times longer compared to the 15 h required by *C. necator* H16 to reach the same OD_600_ 5.0 in gluconate (Figure 1 inset). Lag phase is the most poorly understood growth phase, lacking data that support the underlying physiological and molecular processes [12]. In this case, one could only assume that the long lag phase is required for the bacterial cells to adapt and grow in the new environmental conditions. As shown in Table 1, specific growth rate of *C. necator* H16 in 0.50% (*w*/*v*) glycerol was 0.021 h^−1^, much slower compared to 0.59% (*w*/*v*) sodium gluconate (0.24 h^−1^). The specific growth rates (Table 1) in the different glycerol concentrations investigated were similar (0.020 h^−1^ to 0.026 h^−1^). Unsurprisingly, the maximal achievable OD_600_ values showed a positive correlation with the glycerol concentrations used (6.0 for 0.50%, 13.4 for 1.00%, and 19.9 for 2.00% (*w*/*v*)). The tested range of glycerol concentrations was not reported to be inhibitory for other microorganisms [13,14,15] and increasing the concentration from 0.50% *(w*/*v*) to 2.00% (*w*/*v*) did not show growth inhibition of *C. necator* H16, suggesting the slow growth is likely attributed to slow glycerol metabolism.

### 2.2. Improved Glycerol Utilization in *C. necator* H16 by Adaptive Evolution

Factors limiting microbial growth in glycerol can include the lack of glycerol assimilation pathway [16], lack of gene expression [17] and metabolic pathway regulation [18]. Since the underlying reason of slow *C. necator* H16 growth in glycerol is not understood, adaptive evolution was used to improve the specific growth rate of *C. necator* H16 in glycerol. Adaptive evolution exploits the linkage between carbon (glycerol) utilization and bacterial survival (growth), and was previously applied to different bacteria [19,20].

**Figure 2:**
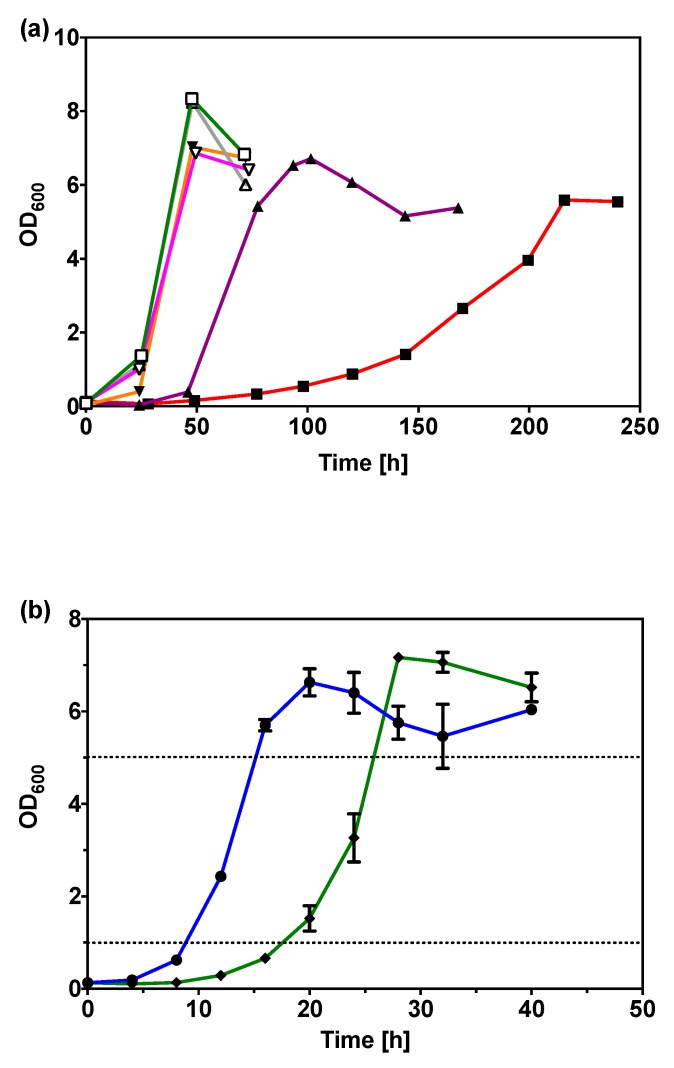
(**a**) Growth curves of *C. necator* H16 in the six serial passages during adaptive evolution; Round 1 (red line; solid square), Round 2 (purple line; solid triangle), Round 3 (orange line; solid inverted triangle), Round 4 (grey line; open triangle), Round 5 (pink line; open inverted triangle), and Round 6 (green line; open square). (**b**) Growth curve of *C. necator* H16 wild-type in 0.59% (*w*/*v*) sodium gluconate (blue line; solid circle) and v6C6 variant in 0.50% (*w*/*v*) glycerol (green line; solid diamond), with the dotted lines indicating OD_600_ values of 1.0 and 5.0.

Mineral salts medium (MSM) [21] with 0.50% (*w*/*v*) glycerol as sole carbon source was used as the initial selective condition in adaptive evolution. The cell population adapted quickly over six serial passages with five to seven generations per cultivation round (Round 1 to Round 6; Figure 2a). Significant improvement in glycerol utilization was obtained, with the time taken to reach maximum optical density decreasing from nine days (Round 1) to four days (Round 2) and to two days (Round 3). The time required to reach maximum optical density did not improve from Round 3 to Round 6, but the lag phase in these rounds was shorter. This metabolic malleability of *C. necator* H16 was also previously demonstrated by its quick adaptation to glucose utilization [22].

From the cell populations in Round 4 to Round 6, 83 single colonies from each round were screened in 96-well microtiter plates. From these, 39 improved variants were rescreened, before the best three variants with similar growth rate were identified as v6C6, v6G7, and v6F8 from Round 6. To ensure the improved phenotype is not a transient adaptation due to variation in gene expression pattern, these three variants were subjected to five consecutive rounds of cultivation in either synthetic medium (MSM with 1% (*w*/*v*) sodium gluconate) or complex medium (NB) in the absence of glycerol. When transferred back to MSM with 0.5% (*w*/*v*) glycerol, these three variants maintained their growth rates at the level when they were first isolated. This showed the improved growth rate in glycerol was a result of genetic modification and not simply a reversible physiological adaptation. Specific growth rate of v6C6 in glycerol was 0.20 h^−1^, 9.5 times the rate of wild-type strain in glycerol, and 83% the rate in sodium gluconate (Table 1). The lag-phase of v6C6 cultivation in 0.50% (*w*/*v*) glycerol remained prominent, taking 17 h to reach OD_600_ of 1.0 and an additional 9 h to reach OD_600_ of 5.0 (late exponential phase) as shown in Figure 2b. This growth rate remained the same, whether the pre-culture was prepared in MSM with 1.00% (*w*/*v*) sodium gluconate or 0.50% (*w*/*v*) glycerol. Further adaptive evolution was performed on v6C6 using 0.25% (*w*/*v*), 2.00% (*w*/*v*) and 5.00% (*w*/*v*) glycerol in MSM followed by screening 267 variants, but it did not yield further improvement (data not shown).

### 2.3. *C. necator* H16 Variant v6C6 Co-Utilizes Glycerol and Gluconate

**Figure 3:**
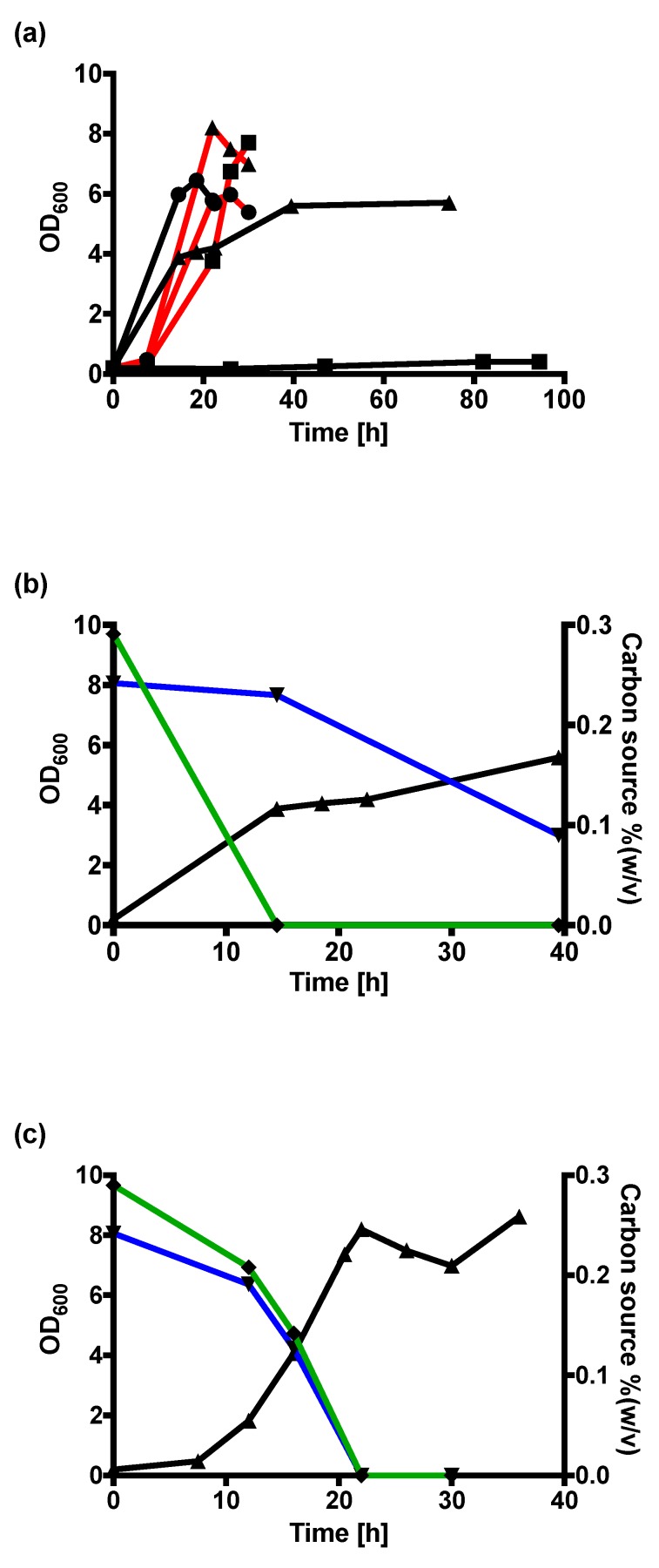
(**a**) Growth curves of wild-type (black lines) and variant v6C6 (red line) in mineral salts medium (MSM) with 0.59% (*w*/*v*) sodium gluconate (circle), MSM with 0.50% (*w*/*v*) glycerol (square), and MSM with 0.30% (*w*/*v*) sodium gluconate and 0.25% (*w*/*v*) glycerol (triangle). (**b**,**c**) The growth curve (black line; triangle) and concentrations of sodium gluconate (green line, diamond) and glycerol (blue line; inverted triangle) in the culture media determined using HPLC for *C. necator* H16 wild-type (b) and v6C6 (c) cultivated in MSM with 0.30% (*w*/*v*) sodium gluconate and 0.25% (*w*/*v*) glycerol.

The co-utilization of gluconate and glycerol by *C. necator* H16 wild-type and variant v6C6 was monitored using HPLC (Figure 3). When cultivated in mixed gluconate–glycerol carbon sources, *C. necator* H16 wild-type uses gluconate and glycerol sequentially (Figure 3b) while variant v6C6 co-utilizes gluconate and glycerol (Figure 3c). Co-utilization enabled v6C6 to achieve a higher optical density in mixed gluconate–glycerol (OD_600_ = 8.2) compared to either gluconate (OD_600_ = 5.97) or glycerol (OD_600_ = 7.7) alone (Figure 3a). Shown in Figure 3b for *C. necator* H16 wild-type, gluconate was completely utilized by 14.5 h and the OD_600_ subsequently increased from OD_600_ = 3.88 (14.5 h) to OD_600_ = 5.70 (74.5 h) through the utilization of glycerol. This contrasted with the OD_600_ < 0.5 after 94.5 h when wild-type was cultivated using only glycerol. It is interesting that the use of gluconate as a co-substrate improved glycerol assimilation, though the preferred gluconate was assimilated first. This observation showed that mixed substrates can be used to enhance glycerol utilization, but it also highlighted the complexity of the regulation mechanism in carbon metabolism. Advantages of using co-substrates with glycerol during fermentation have previously been reported for other organisms. For instance, co-utilization of glycerol and monosaccharides in fermentation of *Clostridium diolis* can increase cell growth and 1,3-propanediol product formation [23]. Similar observations were made for the production of fumaric acid with fungi *Rhizopus oryzae* [24]. These observed improvements had been attributed to various reasons, including the protective role of glycerol under stress conditions [24] and a higher ratio of intracellular NADH/NAD^+^ in co-utilization of glycerol and sugar [23].

### 2.4. Variant v6C6 Has Decreased N-acetylglucosamine and Fructose Utilization

**Figure 4:**
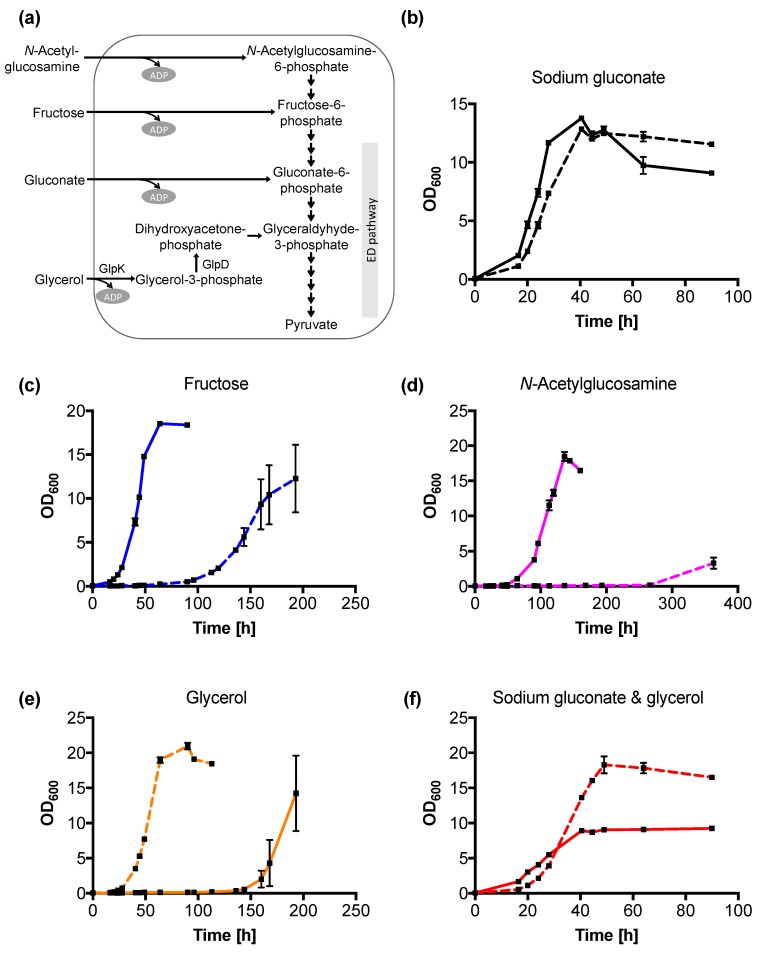
Utilization of different carbon sources by wild-type and variant v6C6. (**a**) The metabolic pathway for *N*-acetylglucosamine, fructose, gluconate, and glycerol assimilation in *C. necator* H16 via the Entner–Doudoroff (ED) pathway. (**b–f**) The growth curves of wild-type (solid line) and v6C6 (dotted line) in MSM with five different carbon sources; 1.0% (*w*/*v*) of sodium gluconate (b), 1.0% (*w*/*v*) fructose (c), 1.0% (*w*/*v*) *N*-acetylglucosamine (d), 1.0% (*w*/*v*) glycerol (e), and 0.5% (*w*/*v*) sodium gluconate and 0.5% (*w*/*v*) glycerol (f).

*N*-acetylglucosamine, fructose, gluconate, and glycerol are catabolized in the Entner–Doudoroff (ED) pathway to yield pyruvate in *C. necator* H16 (Figure 4a). The utilization of the different carbon sources within the ED pathway by v6C6 was investigated in 1.0% (*w*/*v*) of each of these carbon sources as well as in mixed sodium gluconate–glycerol (0.5% (*w*/*v*) each). Excluding the samples with glycerol, both wild-type and v6C6 grew fastest in gluconate, followed by fructose, and finally *N*-acetylglucosamine (Figure 4b–d). While the growth rate of variant v6C6 (0.14 ± 0.01 h^−1^) is similar to wild-type (0.13 ± 0.01 h^−1^) in 1.0% (*w*/*v*) sodium gluconate, its growth in fructose and *N*-acetylglucosamine is significantly slower (Figure 4b–d). In fructose, the wild-type reached stationary phase by 64 h while v6C6 was still in its lag phase (Figure 4c). The same phenomenon was observed for *N*-acetylglucosamine (Figure 4d), where v6C6 was in its lag phase when the wild-type reached stationary phase at 136 h. In media with glycerol, variant v6C6 grew faster than the wild-type (Figure 4e,f), as observed in Figure 3 despite the differences in cultivation conditions (pre-culture prepared in nutrient broth instead of MSM with 1% (*w*/*v*) sodium gluconate, lower starting OD_600_ at 0.04 instead of 0.1, higher concentrations of gluconate and glycerol).

Since mutations occur randomly during adaptive evolution, the cause of significantly slower growth in *N*-acetylglucosamine and fructose is unclear. It was previously shown that the insertion of *glpK_Ec_* to the *C. necator* H16 chromosome improved its glycerol utilization without affecting its growth on fructose [25]. It is thus unlikely that the mutated GlpK in v6C6 is the cause of decreased fructose assimilation. We can only assume that proteins/reactions involved directly or indirectly in the metabolic pathway between *N*-acetylglucosamine-6-phosphate to gluconate-6-phosphate were affected (Figure 4a). Poor assimilation of *N*-acetylglucosamine and fructose can also be due to poorer substrate transport into the cell. However, *C. necator* H16 uses different transport systems for the two substrates; *N*-acetylglucosamine enters the cell via a porin (NagC) in the outer membrane [26] and fructose uptake is mediated by an ABC-type transporter [8].

### 2.5. Variant v6C6 Produced More PHB from Glycerol

**Figure 5:**
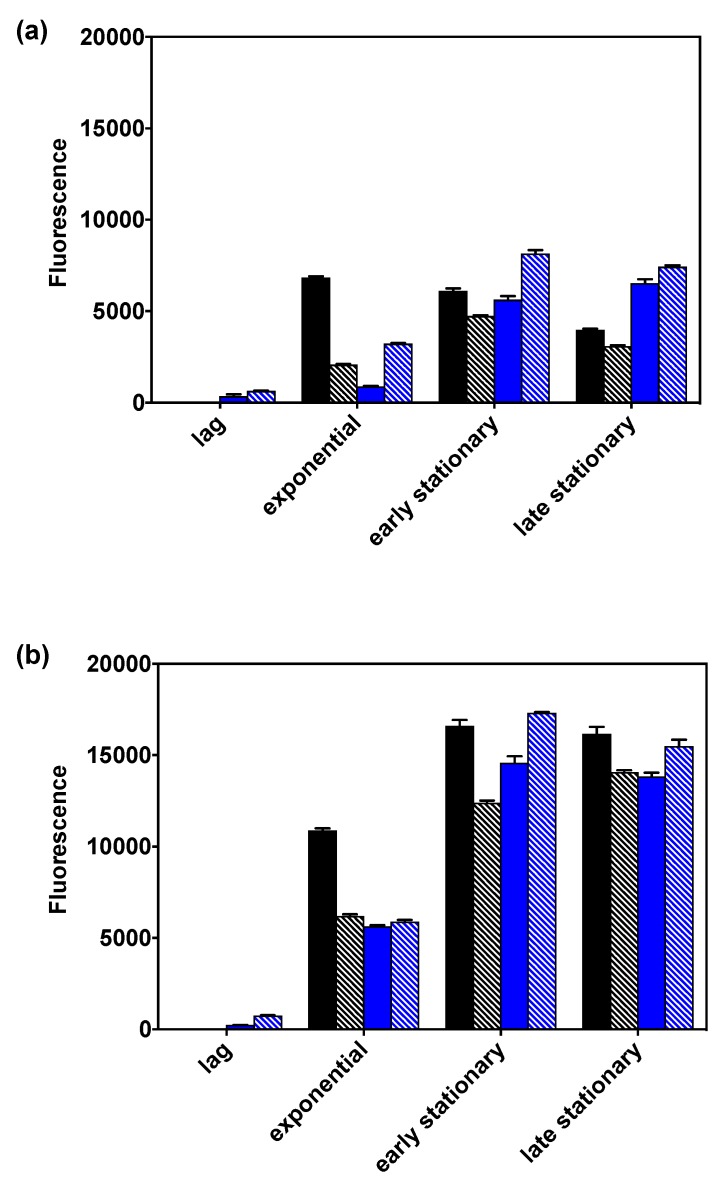
Polyhydroxybutyrate (PHB) production surveyed using the Nile red fluorescence assay for *C. necator* H16 (black solid bars) and v6C6 variant (blue solid bars) in 0.59% (*w*/*v*) gluconate and *C. necator* H16 (black stripe bars) and v6C6 variant (blue stripe bars) in 0.50% (*w*/*v*) glycerol. Cells were cultivated either in nutrient-balanced (**a**) or nitrogen-limiting (**b**) MSM.

PHB productivity of the wild-type and v6C6 in both gluconate and glycerol were surveyed using the Nile red assay [27], a lipophilic stain widely used for PHB quantification (Figure S1). For both strains and in both carbon sources, higher fluorescence was obtained in nitrogen-limiting media (Figure 5), in agreement with the known fact that *C. necator* H16 produces more PHB under nutrient stress [28]. Results also showed that, under nitrogen-limiting conditions, *C. necator* H16 wild-type produced more PHB in gluconate than in glycerol across all growth phases. Interestingly, the reverse is true for the v6C6 variant, where more PHB was produced from glycerol. Maximum PHB production from glycerol was achieved at the early stationary phase in nitrogen-limiting medium for v6C6, and it is 19% more than the PHB produced in the wild-type strain. We further confirmed PHB production through Nile red staining of PHB granules and visualization using fluorescence microscopy (Figure S2).

### 2.6. Variant v6C6 Showed Superior Growth in Crude Glycerol

**Figure 6:**
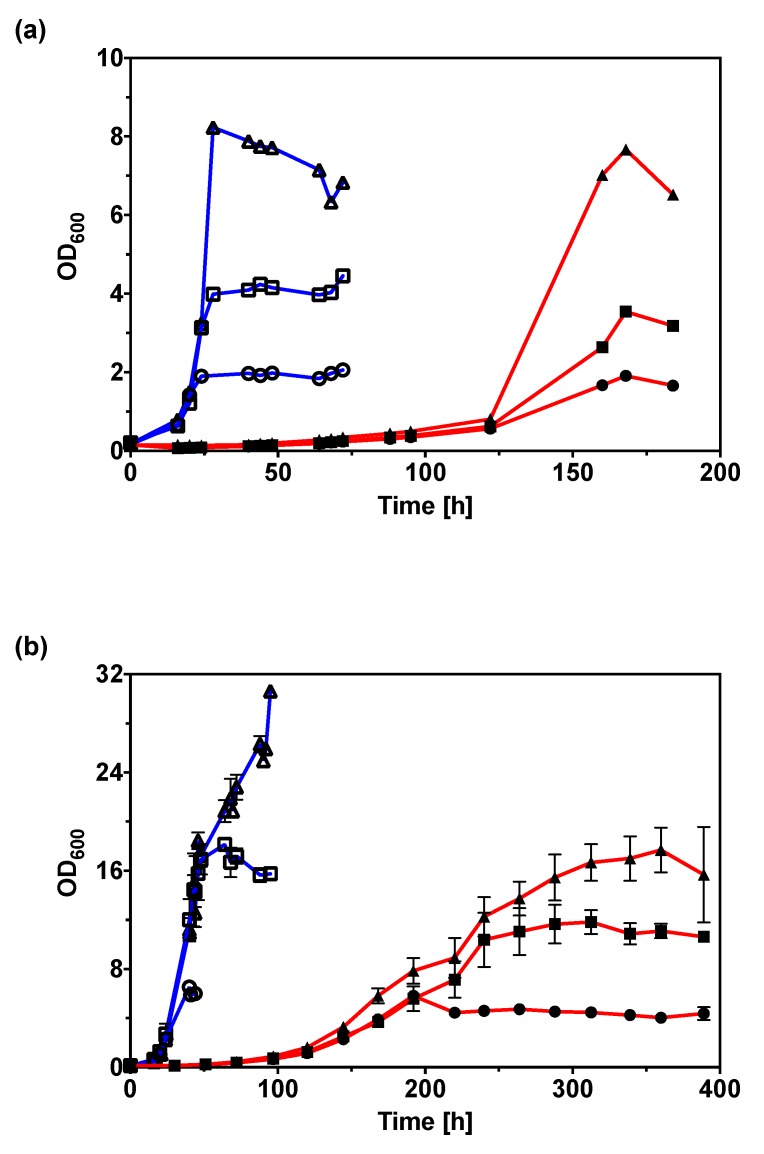
(**a**) Growth of *C. necator* H16 wild-type (red lines, solid symbols) and v6C6 variant (blue lines, open symbols) in 1% (*v*/*v*) (circle), 2% (*v*/*v*) (square), and 4% (*v*/*v*) (triangle) crude glycerol (sweetwater). (**b**) Growth of *C. necator* H16 wild-type (red lines, solid symbols) and v6C6 variant (blue lines, open symbols) in 0.5% (*w*/*v*) (circle), 1% (*w*/*v*) (square), and 2% (*w*/*v*) (triangle) laboratory-grade glycerol.

All aforementioned experiments were conducted using laboratory-grade glycerol with purity over 99%. To verify the potential of the v6C6 variant for industrial application, we conducted comparative growth studies using crude glycerol (commonly known as sweetwater) obtained from a high-pressure fat-splitting process (Figure 6). Though composed of mainly water and glycerol, crude glycerol is a complex mixture that contains impurities such as free fatty acid, unreacted mono-, di-, and tri-glycerides, inorganic salts, and a variety of matter organic non-glycerol (MONG). At all crude glycerol concentrations tested (4% (*v*/*v*), 2% (*v*/*v*), and 1% (*v*/*v*)), v6C6 showed higher specific growth rates and shorter lag phase compared to wild-type (Figure 6a), matching the growth profile observed in laboratory-grade glycerol (Figure 6b). Specific growth rate of v6C6 in 1% to 4% (*v*/*v*) crude glycerol (Table 2) is 0.17 h^−1^ to 0.22 h^−1^, similar to the specific growth rate in 0.5% (*w*/*v*) pure glycerol (0.20 h^-1^).

### 2.7. Genome Sequencing of Improved Glycerol Variants

The genomes of variants v6C6, v6G7, and v6F8 and wild-type were sequenced to identify the number, location, and nature of their genetic modification(s). *C. necator* H16 has two chromosomes and one megaplasmid, with a genome size of 7,416,678 bp. All genomes sequenced have more than 30× mean coverage. Based on the variant calling results of the sequenced genome, a list of four non-synonymous mutations, one insertion, and one deletion were identified against the wild-type genome (Table 3). These four mutations resulted in amino acid changes in the four proteins encoded at the gene loci H16_A0689, H16_A1373, H16_A2507, and H16_A3075 (Table 3). With the exception of H16_A1373, the other mutations are found in all three variants.

Based on the genome annotation (accession number NC_008313, NC_008314, and NC_005241 for chromosome 1, chromosome 2, and megaplasmid pHG1, respectively), the gene encoded by H16_A2507 is predicted to be a glycerol kinase (GlpK) by protein homology. Glycerol kinase is known to participate in the glycerol metabolism by phosphorylating glycerol to glycerol-3-phosphate (Figure 4a). Gene locus H16_A0689 encodes an ornithine cyclodeaminase, an enzyme that directly converts ornithine to proline. H16_A1373 is predicted to be a PAS domain-containing sensor histidine kinase and H16_A3075 encodes a dihydropyrimidinase, a hydrolase that catalyzes the ring-opening of 5,6-dihydrouracil to form 3-ureidopropanoate. It is unclear how these three proteins encoded by H16_0689, H16_1373, and H16_3075 can impact *C. necator* H16 glycerol metabolism or if they have any effect at all.

As a further verification of the mutation in *glpK_v6C6_* gene (gene locus H16_A2507) in v6C6, the gene including its 500-bp upstream element were amplified by PCR and sent for sequencing. Sanger sequencing result of the PCR fragment verified the W480S mutation identified in genome sequencing and no mutations were found in the upstream element where its promoter possibly resides. Being present in all three variants (v6C6, v6G7, and v6F8) suggests the enrichment of W480S mutation during cultivation as it conferred superior growth in glycerol. A conserved domain search [29] revealed that W480 is fairly conserved in a multiple sequence alignment of GlpK_H16_ against related proteins spanning a variety of organisms (Figure S3). Based on the GlpK_H16_ protein model created using GlpK_Ec_ (PDB 1BOT) as a template (Figure S4), W480 resides on a coiled region, away from the catalytic center. As W480 is not on any known activation loop or glycerol-binding and ATP-binding site in GlpK [30,31], it is difficult to predict how or if the mutation resulted in alteration of its catalytic function. Other possibilities are the mutation leads to better expression due to codon change, better/faster protein folding, or better protein solubility within the cellular environment.

**Figure 7:**
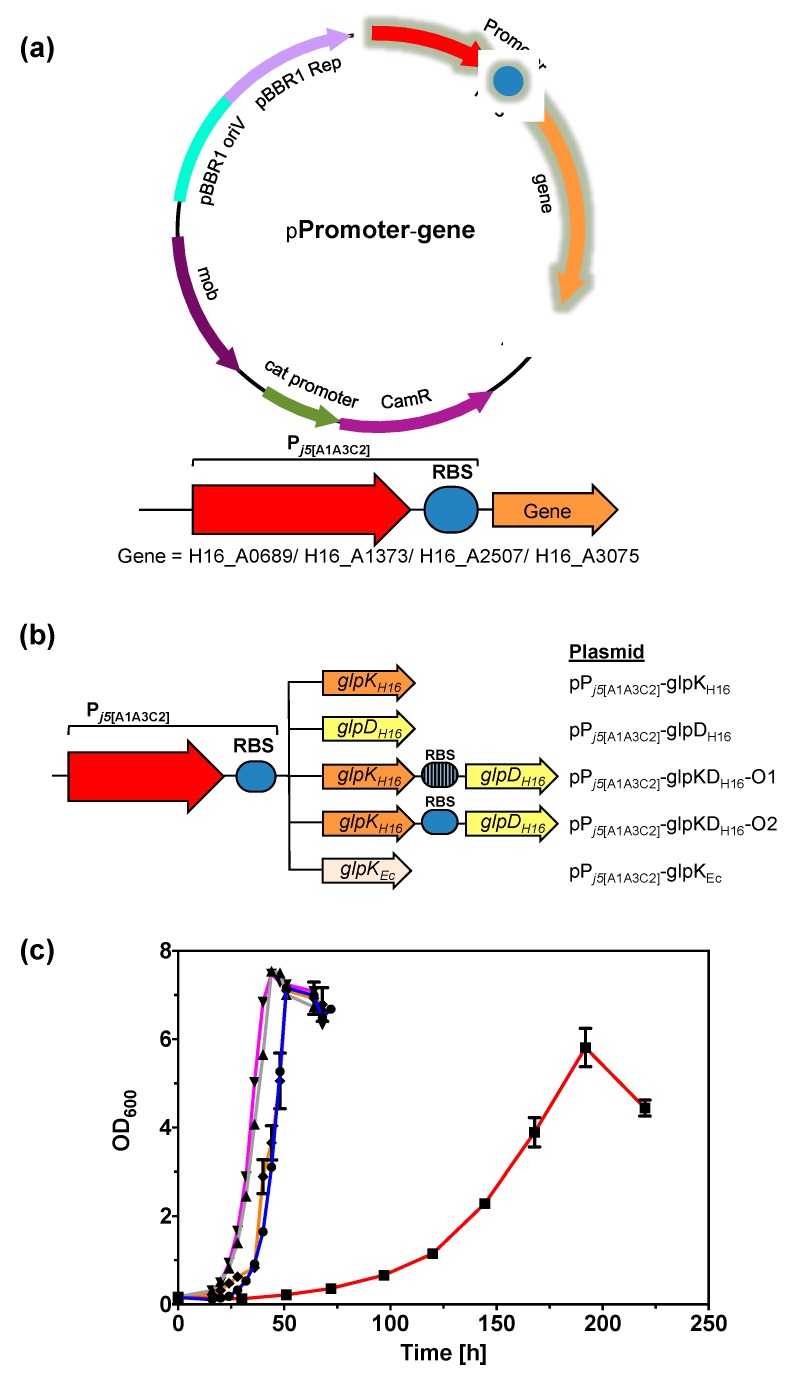
(**a**) General plasmid construct used for cloning of mutated genes identified from genome sequencing. (**b**) Various promoter and gene combinations of *glpK* and *glpD* used in this work. (**c**) Growth curves of *C. necator* H16 harboring *glpK_H16_*, *glpKD_H16_*, and *glpK_Ec_* plasmids when cultivated in 0.50% (*w*/*v*) glycerol; no plasmid (red line; square), pP*_j5_*_[A1A3C2]_-glpK_H16_ (orange line; diamond), pP*_j5_*_[A1A3C2]_-glpKD_H16_-O1 (grey line; triangle), pP*_j5_*_[A1A3C2]_-glpKD_H16_-O2 (pink line, inverted triangle), and pP*_j5_*_[A1A3C2]_-glpK_Ec_ (blue line; circle).

To gain further insights of the four non-synonymous mutations identified in genome sequencing, the four genes were amplified from v6C6 and cloned for constitutive expression in a P*_j5_*-based constitutive plasmids (Figure 7a, Table S1) [10]. The constructs were transformed into *C. necator* wild-type and cultivated in MSM with 1.0% (*w*/*v*) glycerol. Constitutive expression of GlpK_v6C6_ encoded by *glpK_v6C6_* in v6C6 improved growth in MSM with 1.0% (*w*/*v*) glycerol (Figure S5). No difference was, however, observed when the other three protein variants were expressed in the *C. necator* H16 wild-type strain (Figure S5). It is thus likely that GlpK contributed to the improved glycerol assimilation in v6C6. However, expressing GlpK_v6C6_ alone in *C. necator* H16 was not sufficient to reproduce the phenotype of v6C6 (Table 1), suggesting potential synergistic effect of the mutations found in v6C6 variant. It is worth mentioning that expression of *Escherichia coli* GlpK (GlpK_Ec_) in *C. necator* H16 was previously noted to improved glycerol assimilation [25].

### 2.8. Expression of GlpK Enhances the Glycerol Metabolic Pathway

Glycerol is catabolized in the cells by GlpK to glycerol-3-phosphate and then oxidized to dihydroxyacetone-phosphate (DHAP) by glycerol-3-phosphate dehydrogenase (GlpD) before it is metabolized in the sugar-degrading pathway (Figure 4a). The genome of *C. necator* H16 [8] contains two pairs of putative glycerol metabolism genes (GlpK and GlpD; Figure S6 and Table S2). The first pair has the gene loci of H16_A2507 and H16_A2508 and the second pair H16_B1198 and H16_B1199. The GlpK encoded by H16_A2507 has the highest protein sequence identity (52%) to GlpK_Ec_, compared to the other three proteins that have 27% to 30% sequence identity (Table S2). Previous work by Shimizu et al. showed that the first pair of genes had a slightly higher transcriptional level [32].

Since expression of the four mutated genes in v6C6 suggested GlpK may play an important role in glycerol assimilation, the *glpK_H16_* and *glpD_H16_* from gene loci H16_A2507 and H16_A2508 in the wild-type strain were amplified and cloned using pBBR1-based plasmid for expression in *C. necator* H16 wild-type. All genes were cloned downstream of a constitutive promoter P*_j5_*_[A1A3C2]_ [10] (Figure 7b) and individual effects of GlpK_H16_ and GlpD_H16_ expression and in combination (glpKD_H16_) were investigated. Constructs pP*_j5_*_[A1A3C2]_-glpKD_H16_-O1 and pP*_j5_*_[A1A3C2]_-glpKD_H16_-O2 differed in the ribosome binding site (rbs) preceding the *glpD_H16_* gene. While pP*_j5_*_[A1A3C2]_-glpKD_H16_-O1 used the native *glpD_H16_* rbs, pP*_j5_*_[A1A3C2]_-glpKD_H16_-O2 had the same synthetic rbs preceding both *glpK_H16_* and *glpD_H16_* genes. To compare the differences between GlpK from *C. necator* H16 (GlpK_H16_) and GlpK from *E. coli* (GlpK_Ec_), plasmid pP*_j5_*_[A1A3C2]_-glpK_Ec_ was also constructed (Figure 7b).

*Cupriavidus necator* H16 carrying plasmids pP*_j5_*_[A1A3C2]_-glpK_H16_, pP*_j5_*_[A1A3C2]_-glpKD_H16_-O1, or pP*_j5_*_[A1A3C2]_-glpKD_H16_-O2 had specific growth rates of 0.13–0.14 h^−1^ when cultivated in 0.50% (*w*/*v*) glycerol compared to 0.021 h^−1^ for the wild-type (Table 1, Figure 7c). This suggests that glycerol kinase (GlpK_H16_) plays a more dominant role compared to GlpD_H16_ for improving growth in glycerol. Improved glycerol assimilation due to plasmid-based expression of GlpK_H16_ from the wild-type strain also suggested a positive effect of *glpK_H16_* gene dosage on glycerol assimilation. Interestingly, the strain harboring pP*_j5_*_[A1A3C2]_-glpD_H16_ plasmid could not grow in glycerol, even though the presence of plasmid-borne *glpD_H1_*_6_ gene in the transformed cells was confirmed by colony PCR. It is possible that increasing GlpD_H16_ expression leads to DHAP accumulation, which in turn gets converted into methylglyoxal via the action of methylglyoxal synthase (MgsA, encoded by H16_A0932). Methylglyoxal, being a highly reactive dicarbonyl compound, is one of the most potent glycating agents, readily reacting with proteins, lipids, and nucleic acids leading to cellular damage [33]. The *C. necator* H16 transformed with pP*_j5_*_[A1A3C2]_-glpK_Ec_ showed a specific growth rate of 0.15 h^−1^ in glycerol, similar to the improvement achieved with pP*_j5_*_[A1A3C2]_-glpK_H16_ (0.14 h^−1^). Plasmid pP*_j5_*_[A1A3C2]_-glpK_H16_, when transformed into the v6C6 variant, had a specific growth rate of 0.21 h^−1^, similar to the growth rate of v6C6 on its own (0.20 h^−1^), thus no additive effect in growth improvement was observed.

## 3. Methods

### 3.1. Bacterial Strains, Plasmids, and Cultivation Conditions

All bacterial strains and plasmids used in this study are listed in Table 4. *Escherichia coli* strain DH5α, used for molecular cloning and plasmid propagation, was cultivated in 2 × YT medium (16 g/L tryptone, 10 g/L yeast extract, 5 g/L sodium chloride) at 37 °C and 250 rpm (ES-20 shaker-incubator; Grant Instruments, Shepreth, UK). *C. necator* H16 was cultivated in mineral salts medium (MSM, pH 7.0) [34] or nutrient broth (5 g/L peptone, 1 g/L beef extract, 2 g/L yeast extract, 5 g/L sodium chloride) at 30 °C and 250 rpm (MaxQTM 4450 benchtop orbital shaker; Thermo Fisher Scientific, Loughborough, UK). Stock solution of glycerol was sterilized either by autoclave or using a 0.2 μm filter. When necessary, the medium was supplemented with 25 μg/mL chloramphenicol. Further, 10 μg/mL gentamicin was always added for the cultivation of *C. necator* H16 and its variants. Cell growth was monitored via optical density measurement at 600 nm (BioPhotometer Plus UV/Vis photometer; Eppendorf, Stevenage, UK) or at 595 nm (MultiskanTM FC microplate photometer; Thermo Fisher Scientific, Loughborough, UK). Specific growth rate was calculated by fitting the exponential growth to the exponential growth equation (Y = Y0 × e^kX^) provided in GraphPad Prism (GraphPad Software, La Jolla, CA, USA).

### 3.2. Adaptive Evolution of *C. necator* H16 for Enhanced Growth in Glycerol

Adaptive evolution of *C. necator* H16 was performed by serial passages in 5 mL of MSM supplemented with 0.50% (*w*/*v*) glycerol as sole carbon source. In each round, cells were grown to early stationary phase (OD_600_ 5−6) before 50 μL was used to inoculate the subsequent round. Serial passage was continued until there was no significant improvement in cell growth rate. A glycerol stock of the cell population in each round was prepared and stored at −80 °C. Cells from the fourth to sixth rounds were re-cultivated in MSM with 0.50% (*w*/*v*) glycerol to OD_600_ 1.0 before they were plated on MSM agar plate with the same glycerol concentration. Agar plates were incubated at 30 °C for 48 h before ~90 single colonies from each round were transferred to 96-well microtiter plates and re-screened in MSM with 0.50% (*w*/*v*) glycerol. The fastest growing variants were isolated for characterization.

### 3.3. Confirmation of Improved Glycerol-Utilizing Phenotype

Improved glycerol-utilizing phenotype in the identified variants was verified by five rounds of cultivation, in either MSM with 1.00% (*w*/*v*) sodium gluconate or nutrient broth (Figure S7). In each of these rounds, cells were cultivated for 48 h at 30°C. Cells from the fifth round were then used to inoculate MSM with 0.50% (*w*/*v*) glycerol. True variants were expected to grow as quickly as when they were first isolated, in contrast to those that exhibited transient adaptation.

### 3.4. Carbon Utilization of *C. necator* H16 Wild-Type and v6C6 in Gluconate–Glycerol Medium

Pre-cultures of *C. necator* H16 wild-type and variant v6C6 were prepared from single colonies in MSM with 1.00% (*w*/*v*) sodium gluconate. Each pre-culture was used to inoculate 5 mL MSM with different carbon sources to a starting OD_600_ of 0.2. Three different media were investigated; MSM with 0.60% (*w*/*v*) sodium gluconate, MSM with 0.50% (*w*/*v*) glycerol, and MSM with 0.30% (*w*/*v*) sodium gluconate and 0.25% (*w*/*v*) glycerol. Culture (0.5 mL) was sampled at start of cultivation, log phase, and stationary phase of growth. Sample was centrifuged (21,000 g, 5 min) to separate the cells from the spent culture media. The spent media was clarified using a 0.2-μm spin column (10,000 g, 1 min) to remove any cell particles. Concentrations of sodium gluconate and glycerol in the culture media were determined by high-performance liquid chromatography (HPLC) using the Prominence-i LC-2030C Plus (Shimadzu UK Ltd., Milton Keynes, UK) equipped with a Rezex ROA-Organic Acid column (300 × 7.8 mm; Phenomenex, Macclesfield, UK) and a refractive index detector. Ten microliters of sample were injected using an autosampler and isocratic separation of sodium gluconate and glycerol was achieved at 60 °C using 0.005 N sulphuric acid flowing at 0.6 mL/min as the mobile phase. The concentrations of these compounds were estimated from standard curves generated by analyzing known concentrations of sodium gluconate and glycerol (both ≥  99% purity; Sigma-Aldrich, Dorset, UK). The standard error of the HPLC measurements was always between 1.0%–1.5%.

### 3.5. Growth of *C. necator* H16 Wild-Type and v6C6 in Different Carbon Sources

Pre-culture of *C. necator* H16 WT and v6C6 was cultivated from a single colony in 5 mL of nutrient broth at 30°C. Fifty microliters of pre-culture were used to inoculate MSM with five different carbon sources (1.0% (*w*/*v*) sodium gluconate, 1.0% (*w*/*v*) fructose, 1.0% (*w*/*v*) *N*-acetylglucosamine, 1.0% (*w*/*v*) glycerol, or 0.5% (*w*/*v*) each of gluconate and glycerol). Cell growth was monitored via optical density measurement at 600 nm. The experiment was performed in triplicate.

### 3.6. Analysis of PHB Content Using the Nile Red Assay

*C. necator* H16 wild-type and its v6C6 variant were each cultivated in four different media; (i) MSM with 0.59% (*w*/*v*) gluconate and 0.10% (*w*/*v*) NH_4_Cl; (ii) MSM with 0.59% (*w*/*v*) gluconate and 0.05% (*w*/*v*) NH_4_Cl; (iii) MSM with 0.50% (*w*/*v*) glycerol and 0.10% (*w*/*v*) NH_4_Cl; and (iv) MSM with 0.50% (*w*/*v*) glycerol and 0.05% (*w*/*v*) NH_4_Cl. Half a milliliter of pre-culture was used to inoculate 50 mL of each medium. The cells were cultivated at 30 °C and 250 rpm, and the growth was monitored using optical density measurement. Cell samples were taken at the lag, exponential, early stationary, and late stationary phases for PHB quantification. When OD_600_ > 2, the cell samples were diluted to OD_600_ ≤ 2 using MSM to keep the fluorescence detection within the linear range (Figure S1). Subsequently, 1-mL aliquots of all cells were centrifuged and spent media was removed. Cell pellets were stored at −20 °C until all samples were collected. To compare PHB production using the Nile red assay, all cell pellets were thawed, and each was re-suspended in 0.5 mL of 50% (*v*/*v*) ethanol solution before 50 μL of cell suspension was mixed with 50 μL of 10 µg/mL Nile Red in a microtiter plate. The fluorescence was measured at an excitation wavelength of 552 nm and an emission wavelength of 600 nm using the SpectraMax M2e microplate reader (Molecular Devices, Winnersh, UK) for 1 h at 10 min intervals. Fluorescence values were stable at 1 h for all samples and comparisons between samples were made at this time point. The experiment was performed in triplicate. Comparison of PHB production was based on the fluorescence per 100 μL of cell culture.

### 3.7. Growth of *C. necator* H16 in Crude Glycerol (Sweetwater)

Crude glycerol (sweetwater) sample (batch 8/1/18; 10–15% *v*/*v* glycerol), obtained from a high-pressure fat-splitting process, was kindly provided by Croda (Hull, UK). A pre-culture was prepared from a single colony in MSM with 1.00% (*w*/*v*) gluconate. For growth comparison, 5 mL of MSM with 1.00%–4.00% (*v*/*v*) crude glycerol was inoculated to a starting OD_600_ of 0.1 using the pre-culture. Cells were cultivated at 30 °C and their optical density monitored.

### 3.8. Genome Sequencing

The strains (wild-type, v6C6, v6F8, and v6G7) were streaked out on a nutrient broth agar plate with gentamicin and incubated at 30 °C for 40−48 h. A single colony was isolated and resuspended in 100 μL of sterile PBS buffer. The resuspended cells are then streaked out on a fresh nutrient broth agar plate with gentamicin again to create a bacteria lawn that covers about a third of the plate and cultivated at 30 °C for 40−48 h. A sterile loop was used to transfer all the bacteria from the plate into the barcoded bead tube provided by MicrobesNG (Birmingham, UK). Genomic DNA extraction, genome sequencing, genome assembly, and variant calling were performed by MicrobesNG.

### 3.9. Expression of GlpK and/or glpD Genes in *C. necator* H16–Plasmid Construction and Bacterial Cultivation

Total genomic DNA of *E. coli* BW25113, *C. necator* H16 wild-type, and *C. necator* H16 variant v6C6 were isolated using E.Z.N.A.^®^ Bacterial DNA Kit (Omega Bio-Tek, Norcross, GA, USA). Genes *A0689_v6C6_*, *A1373_v6C6_*, H16, *glpK_v6C6_*, and *A3075_v6C6_* were amplified from the v6C6 variant by PCR. Genes *glpK_H16_*, *glpD_H16_*, and *glpK_Ec_* were amplified from the wild-type strain of the respective genomic DNA by PCR. All DNA modifying enzymes were purchased from New England Biolabs Ltd. (Hitchin, UK) and used according to the manufacturer’s instruction. All oligonucleotides were purchased from Eurofins Genomics (Ebersberg, Germany). The sequences of all primers used in this work are shown in Table S3. Plasmid DNA was isolated using E.Z.N.A.^®^ Plasmid Mini Kit I (Omega Bio-Tek, Norcross, GA, USA). PCR and gel purifications were performed using NucleoSpin^®^ Gel and PCR Clean-up (Macherey-Nagel, Düren, Germany). All genes were cloned by replacing the rfp gene in pP*_j5_*_[C2]_-RFP [10] with the target gene. Standard restrictive digestion and ligation were used for all plasmids except pP*_j5_*_[A1A3C2]_-glpKD_H16_-O2, which was constructed using the NEBuilder^®^ HiFi DNA Assembly Cloning Kit (New England Biolabs Ltd., Hitchin, UK). Plasmids were transformed into *E. coli* DH5α using the standard CaCl_2_ method. DNA sequencing showed mutations in the promoter region of pP*_j5_*_[C2]_-glpK_H16_. For this reason, all genes were subsequently re-cloned to have the same mutated promoter (P*_j5_*_[A1A3C2]_) for a fair comparison (see Table S1 for the sequence of P*_j5_*_[A1A3C2]_).

To determine their effects on growth in glycerol, plasmids were transformed into *C. necator* H16 by electroporation [36]. A pre-culture was prepared from a single colony in MSM with 1.00% (*w*/*v*) gluconate. For growth comparison, 5 mL of MSM with 0.50% (*w*/*v*) glycerol was inoculated to a starting OD_600_ of 0.1 using the pre-culture unless otherwise stated. Cells were cultivated at 30 °C and their optical density monitored. The experiment was performed in triplicate. *C. necator* H16 wild-type or its v6C6 variant was always included for comparison.

## 4. Conclusions

This work unlocked the glycerol-utilizing ability of *C. necator* H16 through the use of adaptive evolution. The best variant, v6C6, has a specific growth rate that is 9.5 times faster than the wild-type, co-utilizes gluconate and glycerol, and produces more PHB in glycerol compared to its favorable carbon source of gluconate. Whole-genome sequencing of the wild-type and three variants identified four non-synonymous mutations in the genome and provided insights into the genetic changes that led to the improved glycerol-utilizing phenotype observed. Expression of the proteins encoded by these mutated genes in v6C6 suggested GlpK_H16_ has an important role in this improved glycerol assimilation. Further investigation through plasmid-based expression of the GlpK and GlpD in the glycerol metabolic pathway confirms the effect of GlpK expression for improving glycerol metabolism in *C. necator* H16. Importantly, the improved glycerol assimilation of v6C6 was also demonstrated for sweetwater, a glycerol-rich waste stream obtained from the industrial fat-splitting process. Improved glycerol utilization and the ability to co-utilize glycerol with other carbon sources in variant v6C6 presents the opportunity for economical polyhydroxyalkanoate production from industrial glycerol by-product and other carbon waste feedstock.

## Figures and Tables

**Table 1:** Specific growth rate of *C. necator* H16 and v6C6 variant in sodium gluconate and laboratory-grade glycerol.

Strain	Plasmid	Carbon Source	Specific Growth Rate (h^−1^)
H16	n.a.	0.59% (*w*/*v*) sodium gluconate	(2.4 ± 0.2) × 10^−1^
H16	n.a.	0.5% (*w*/*v*) glycerol	(2.1 ± 0.1) × 10^−2^
H16	n.a.	1.0% (*w*/*v*) glycerol	(2.0 ± 0.2) × 10^−2^
H16	n.a.	2.0% (*w*/*v*) glycerol	(2.6 ± 0.2) × 10^−2^
v6C6	n.a.	0.5% (*w*/*v*) glycerol	(2.0 ± 0.1) × 10^−1^
v6C6	n.a.	1.0% (*w*/*v*) glycerol	(1.8 ± 0.1) × 10^−1^
H16	pP*_j5_*_[A1A3C2]_-glpK_v6C6_	1.0% (*w*/*v*) glycerol	(0.8 ± 0.0) × 10^−1^
H16	pP*_j5_*_[A1A3C2]_-glpK_H16_	0.5% (*w*/*v*) glycerol	(1.4 ± 0.1) × 10^−1^
H16	pP*_j5_*_[A1A3C2]_-glpD_H16_	0.5% (*w*/*v*) glycerol	-
H16	pP*_j5_*_[A1A3C2]_-glpKD_H16_-O1	0.5% (*w*/*v*) glycerol	(1.3 ± 0.0) × 10^−1^
H16	pP*_j5_*_[A1A3C2]_-glpKD_H16_-O2	0.5% (*w*/*v*) glycerol	(1.4 ± 0.0) × 10^−1^
H16	pP*_j5_*_[A1A3C2]_-glpK_Ec_	0.5% (*w*/*v*) glycerol	(1.5 ± 0.1) × 10^−1^
v6C6	pP*_j5_*_[A1A3C2]_-glpK_H16_	0.5% (*w*/*v*) glycerol	(2.1 ± 0.1) × 10^−1^

**Table 2:** Specific growth rate of *C. necator* H16 and v6C6 variant in crude glycerol (sweetwater).

Strain	Crude Glycerol (% (v/v))	Specific Growth Rate (h^−1^)
H16	1	(2.3 ± 0.1) × 10^−2^
H16	2	(3.1 ± 0.2) × 10^−2^
H16	4	(4.8 ± 0.4) × 10^−2^
v6C6	1	(1.7 ± 0.5) × 10^−1^
v6C6	2	(2.2 ± 0.3) × 10^−1^
v6C6	4	(2.2 ± 0.1) × 10^−1^

**Table 3:** Mutations identified in variants v6C6, v6F8, and v6G7. Nucleotide changes were identified by comparison to the sequenced wild-type.

Chromosome	Gene Position	Mutation	Amino Acid Substitution	Strains with Mutation	Locus Tag	Product or Function (Protein ID)
1	738,900	T→A	M129K	v6C6 v6F8 v6G7	H16_A0689	Ornithine cyclodeaminase (WP_010813066.1)
1	1,486,642	G→C	S736R	v6C6	H16_A1373	Histidine kinase (WP_037024292.1)
1	1,487,637	Deletion (2 bp)	n.a.	v6F8 v6G7	H16_A1373	Histidine kinase (WP_037024292.1)
1	1,864,239	Insertion (42 bp)	n.a.	v6F8 v6G7	n.a.	n.a.
1	2,723,382	G→C	W480S	v6C6 v6F8 v6G7	H16_A2507	Glycerol kinase (WP_011615701.1)
1	3,328,089	A→C	N261K	v6C6 v6F8 v6G7	H16_A3075	Dihydropyrimidinase (WP_041687507.1)

**Table 4:** Bacterial strains and plasmids used in this study.

Bacterial Strains and Plasmids	Description	Reference or Source
**Bacterial Strains**		
***E. coli* DH5α**	Standard cloning strain	Lab collection
***E. coli* BW25113**	Wild-type	[35]
***C. necator* H16**	Wild-type	DSM 428
**v6C6**	Variant of *C. necator* H16 with improved glycerol utilization	This study
**Plasmids**		
**pBBR1c-RFP**	Cam^R^, mob, pBBR1 Rep, pBBR1 oriV, *araC*, P*_BAD_*, *rfp*	This study
**p** **P*_j5_*_[C2]_-** **RFP**	Cam^R^, mob, pBBR1 Rep, pBBR1 oriV, P*_j5_*_[C2]_, *rfp*	This study
**pP*_j5_*_[A1A3C2]_** **-glpK_Ec_**	Cam^R^, mob, pBBR1 Rep, pBBR1 oriV, P*_j5_*_[A1A3C2]_, *glpK_Ec_*	This study
**pP*_j5_*_[A1A3C2]_** **-glpK_H16_**	Cam^R^, mob, pBBR1 Rep, pBBR1 oriV, P*_j5_*_[A1A3C2]_, *glpK_H16_*	This study
**pP*_j5_*_[A1A3C2]_** **-glpD_H16_**	Cam^R^, mob, pBBR1 Rep, pBBR1 oriV, P*_j5_*_[A1A3C2]_, *glpD_H16_*	This study
**pP*_j5_*_[A1A3C2]_** **-glpKD_H16_-O1**	Cam^R^, mob, pBBR1 Rep, pBBR1 oriV, P*_j5_*_[A1A3C2]_, *glpKD_H16_*, native *glpD_H16_* rbs preceding *glpD_H16_*	This study
**pP*_j5_*_[A1A3C2]_** **-glpKD_H16_-O2**	Cam^R^, mob, pBBR1 Rep, pBBR1 oriV, P*_j5_*_[A1A3C2]_, *glpKD_H16_*, synthetic rbs preceding *glpD_H16_*	This study

## Supplementary Materials

Supplementary materials can be found at https://www.mdpi.com/1422-0067/20/22/5737/s1. References [37] are cited in the supplementary materials.

## Abbreviations

PHB	polyhydroxybutyrate
MSM	mineral salts medium
GlpK	glycerol kinase
GlpD	glycerol dehydrogenase
rbs	ribosome binding site
DHAP	dihydroxyacetone-phosphate
