# Supplementary material for: Adaptive Laboratory Evolution of Cupriavidus necator H16 for Carbon Co-Utilization with Glycerol
Source: Int J Mol Sci. 2019 Nov 15;20(22):5737. doi: 10.3390/ijms20225737 (PMC6888959; doi:10.3390/ijms20225737)
Supplement: Supplementary file 1 [file ijms-20-05737-s001.pdf]

Supplementary material

Title

Adaptive laboratory evolution of *Cupriavidus necator* H16 for carbon co-utilization with glycerol

Short title

Glycerol and gluconate co-utilization in *C. necator* H16

Miriam González-Villanueva[1], Hemanshi Galaiya[1], Paul Staniland[2], Jessica Staniland[2], Ian Savill[2], Tuck Seng Wong[1,3*], Kang Lan Tee[1*]

[1]Department of Chemical & Biological Engineering and Advanced Biomanufacturing Centre, University of Sheffield, Sir Robert Hadfield Building, Mappin Street, Sheffield S1 3JD, United Kingdom. [2]Croda Europe Ltd, Oak Road, Clough Road, Hull HU6 7PH, United Kingdom. [3]National Center for Genetic Engineering and Biotechnology, 113 Thailand Science Park, Phahonyothin Road, Khlong Luang, Pathum Thani 12120, Thailand.

*Address correspondence to:

Dr. Kang Lan Tee
Email: k.tee@sheffield.ac.uk
Tel: +44 (0)114 222 7507
Fax: +44 (0)114 222 7501

Or

Dr. Tuck Seng Wong
Email: t.wong@sheffield.ac.uk
Tel: +44 (0)114 222 7591
Fax: +44 (0)114 222 7501

A. Materials and Methods

Protein model of GlpK

Protein model of GlpK from *C. necator* H16 and v6C6 variant were generated using SWISS-MODEL [37], with $GlpK_{Ec}$ from *E. coli* (PDB code 1BOT) as a template protein. Graphics were generated using PyMOL (The PyMOL Molecular Graphics System, Version 2.0 Schrödinger, LLC).

Fluorescence microscopy of Nile red-stained PHB

Samples of *C. necato*r H16 and v6C6 variant cultivated to early-stationary phase in sodium gluconate or glycerol under nitrogen-limiting conditions were used. Samples were adjusted to an OD_{600} of 7.0 before 200 µL-cell aliquots were centrifuged and spent media removed. The cell pellets were re-suspended in 200 µL of 50% (v/v) ethanol before mixing with an equal volume of Nile red (10 µg/mL). Samples were incubated in the dark for 10 min. Cells were centrifuged and supernatant removed. The cell pellets were then re-suspended in 50 µL of PBS buffer and used for fluorescence microscopy. Microscope used was RX30F (Brunel Microscope Ltd, Chippenham, UK) and cells were visualized using filter G-1 (Ex 560 nm | Em 645 nm).

B. Tables and Figures

Table S1: Comparison of $P_{j5[A1A3C2]}$ and $P_{j5[C2]}$ promoter sequences. The red boxes highlight the differences between the 2 promoters.

Promoter	Sequence (5' to 3')
$P_{j5[C2]}$	agcggatataaaaaccgttattgacacaggtggaaatttagaatatactgttagtaaacctaatggatcgaccttagatcttttaagaaggagatatacat
$P_{j5[A1A3C2]}$	agcggatataaaaaccgttattgacacaggtggaaatttagaatatacggttagtaaacctaatggatcgacctagatcttttaagaaggagatatacat

Table S2: Sequence identity between potential glycerol metabolism enzymes in *C. necator* H16 and $GlpK_{Ec}$ and $GlpD_{Ec}$ in *Escherichia coli*.

Gene ID	Original annotation	Identity to *E. coli* protein (% / overlap)
H16_A2507	Glycerol kinase	$GlpK_{Ec}$ (52 % / 501 aa)
H16_A2508	Glycerol-3-phosphate dehydrogenase	$GlpD_{Ec}$ (29 % / 460 aa)
H16_B1198	FAD-dependent glycerol-3-phosphate dehydrogenase	$GlpD_{Ec}$ (27 % / 464 aa)<br>$GlpA_{Ec}$ (30 % / 395 aa)
H16_B1199	Glycerol kinase	$GlpK_{Ec}$ (28 % / 507 aa)

Adapted from Fukui *et al.* [25].

Table S3: All primers used in this work.

Name	Sequence (5' to 3')	Purpose
vReH16-glpK-F	attcgcgcaccagcgtcgccagcggatc	Amplify H16_A2507 with its 500-bp upstream element
vReH16-glpK-R	gccgccgccgatgacgatcacatcc	Amplify H16_A2507 with its 500-bp upstream element
NdeI-H16_A0689-F	ggaattccatatgaaaagcaccgcccgatccctg	Amplify H16_A0689
XhoI-H16_A0689-R	aacgctcgagtttcaggccactgccgcctg	Amplify H16_A0689
NdeI-H16_A1373-F	ggaattccatatgccgtttttcgaccgac	Amplify H16_A1373
XhoI-H16_A1373-R	aacgctcgagttattaatgctcggccagc	Amplify H16_A1373
NdeI-glpk-F	ggaattccatatgaaccagccagcc	Amplify H16_A2507 and $glpKD_{H16}$
XhoI-H16-A2507-R	aacgctcgagttatcaggcgtggctgccgctg	Amplify H16_A2507
NdeI-H16_A3075-F	ggaaggccatatggtgaaacagttcgacctg	Amplify H16_A3075
XhoI-H16_A3075-R	aacgctcgagttatcacaggctgccgcc	Amplify H16_A3075
BW25113_glpk-F	gctccatatgactgaaaaaaaatatatcg	Amplify $glpK_{Ec}$
BW25113_glpk-R	tataatcctcgagttattcgtcgtgttc	Amplify $glpK_{Ec}$
NdeI-glpD-f	ggaattcatatgcagagagtctcc	Amplify $glpD_{H16}$
XhoI-glpD-R	ttaactcgagttatcagccgagcatgta	Amplify $glpD_{H16}$
RBS-glpD-F	agatcttttaagaaggagatatacatatgcagagagtctc	Amplify $glpKD_{H16}$ with a synthetic rbs added
Hifi-F	tacatgctcggctgataactcgagtaaggat	Vector amplification for cloning $glpKD_{H16}$ with synthetic rbs
Hifi-R	cttcttaaaagatcttcaggcgtggctgcc	Vector amplification for cloning $glpKD_{H16}$ with synthetic rbs

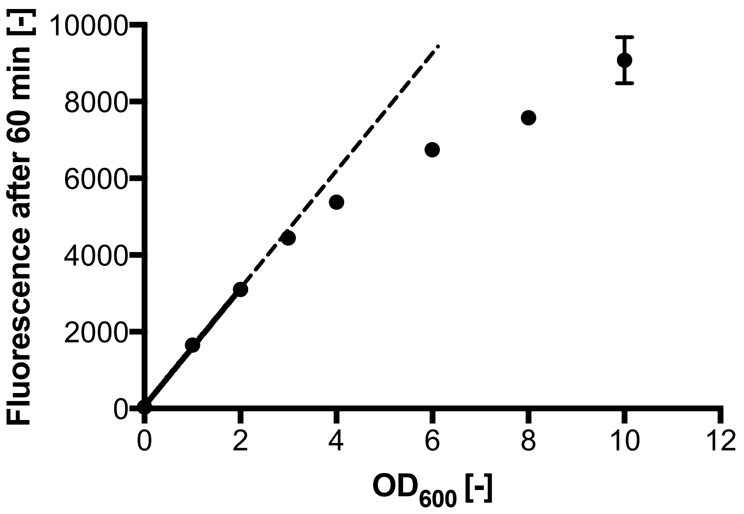

Figure S1: Standard curve of Nile red assay for PHB quantification.

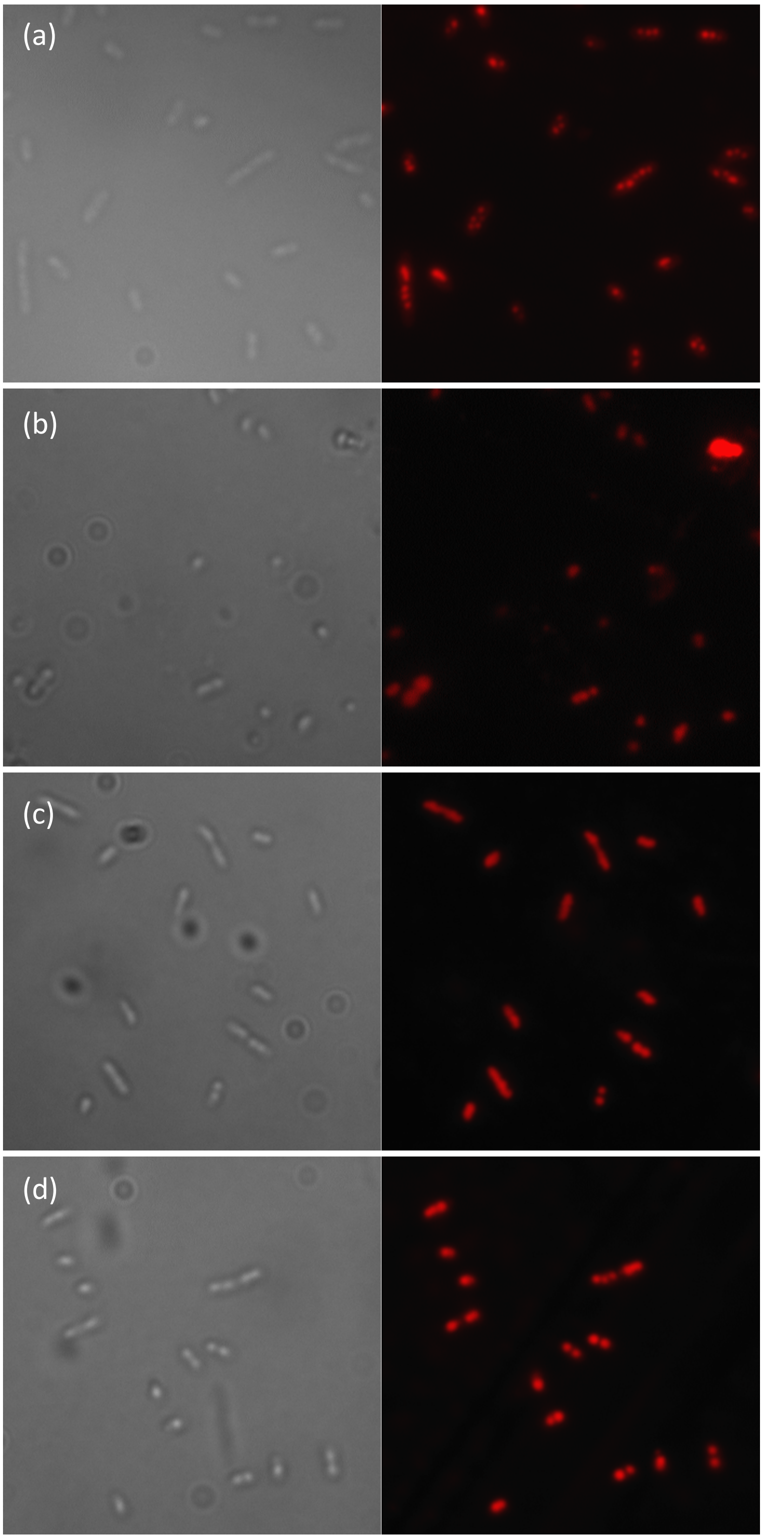

Figure S2: Fluorescence microscopy of Nile red-stained PHB granules. (**a**) *C. necator* H16 cultivated in gluconate under nitrogen-limiting condition (bright field/red channel). (**b**) *C. necator* H16 cultivated in glycerol under nitrogen-

limiting condition (bright field/red channel). (**c**) v6C6 variant cultivated in gluconate under nitrogen-limiting condition (bright field/red channel). (**d**) v6C6 variant cultivated in glycerol under nitrogen-limiting condition (bright field/red channel).

```
                           W480
                             ↓
GlpKH16        475 EIAQQWQVERRFEPnLSADARGHRLARWHRAVD 507
1GLF_Y         459 ELQEKAVIEREFRPgIETTERNYRYAGWKKAVK 491
1BWF_Y         459 ELQEKAVIEREFRPgIETTERNYRYAGWKKAVK 491
1GLD_G         459 ELQEKAVIEREFRPgIETTERNYRYAGWKKAVK 491
1BO5_O         459 ELQEKAVIEREFRPgIETTERNYRYAGWKKAVK 491
2ZF5_O         450 EIAELWKAERIFEPkMDEKTRERLYKGWKEAVK 482
gi 24636875    458 EIKQKWVLDKEFTPnMPKEERDKKYAGWLKAVE 490
gi 219676384   459 EIKRIWQPEREFSVgMSAAARRRRMQSWQAAVD 491
gi 10954725    459 TFAANWKAKRSFTPnLNTDERARRYRKWRAAVA 491
gi 162455899   483 DAARMSRVGQSFQVeMSEAARSAHLHRWADAVA 515
gi 166232274   458 VIAKMPVEQKRFEPrMGSAEALALRHQWEKALS 490
gi 171911423   459 EISTQWQVERTFEPkMPKARVQELRSRWNQALG 491
gi 33866485    462 IAAHRQDDVQRFEPqINTERRRLQRDRWNDAVN 494
gi 6685462     457 EIVSLWQVDKIFEPsMPKNQKEKLLENWNKAVG 489
gi 225026325   456 EIKELWEADRVFEPqMDEETRENLYAQWCKAVE 488
gi 221126353   460 ELTDLWKAERRFLPtLPPARAKELMERWEHAVR 492
gi 94495920    453 DAAAAMPIRQRFKPrMTAPTRAARLGNWNDILR 485
gi 46576631    456 ELRSIWKVDKEFIPsMSEEKRRALYSGWKEAVK 488
gi 166990495   460 ELKEQWCLDKVFNPqMKEDTARKLLNEWHKAVG 492
gi 68052088    469 ELRDNWQVDEEFSPeMDAGKADKMYARWDDAVD 501
```

Figure S3: Conserved Domain Search [29] of *C. necator* H16 $GlpK_{H16}$. Figure shows multiple sequence alignment of $GlpK_{H16}$ against related proteins from a variety of organisms and the protein sequences used to curate the domain model. Sequence alignment is shown using colour bits 3.5 for the amino acids, where red indicates highly conserved and blue indicates less conserved.

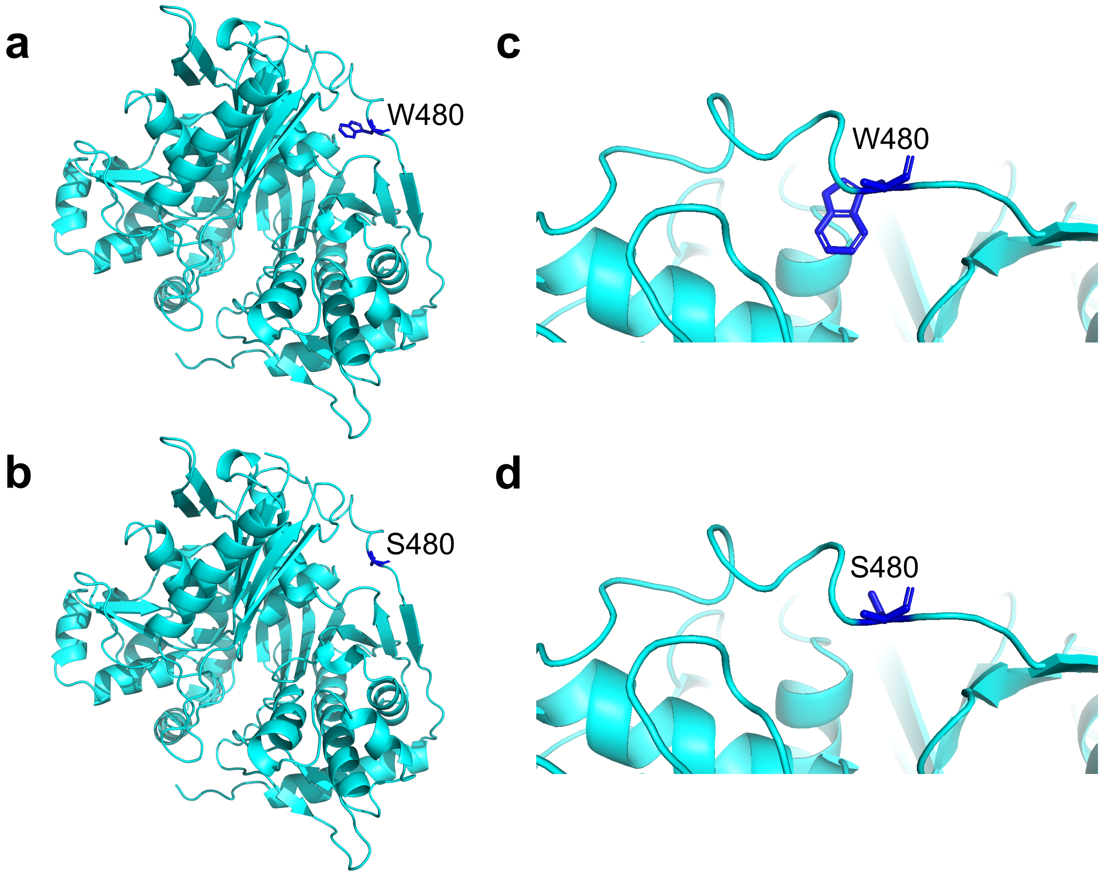

Figure S4: Protein models of *C. necator* H16 GlpK (gene locus: H16_A2507). (**a** & **c**) GlpK wild type with Trp480 highlighted in blue. (**b** & **d**) GlpK W480S mutant with Ser480 highlighted in blue.

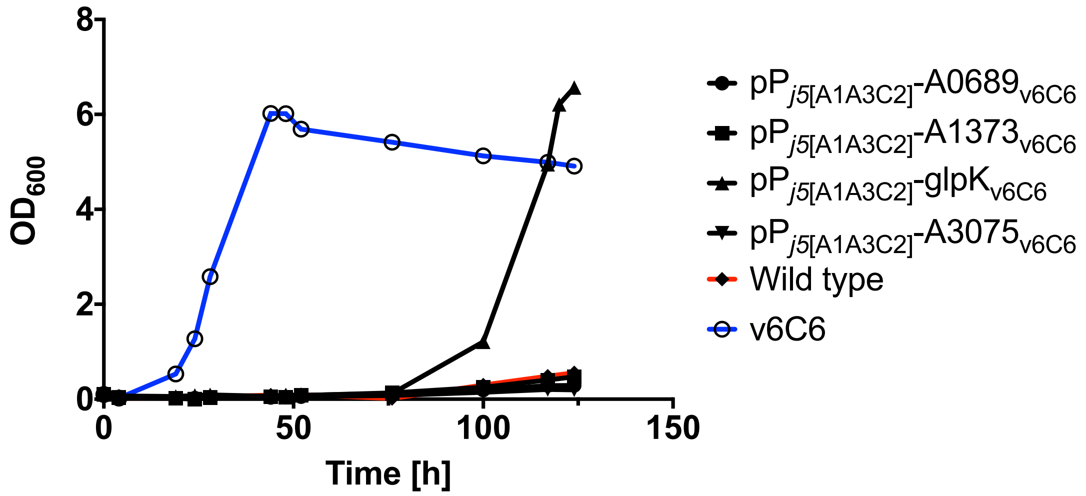

Figure S5: Growth curve of *C. necator* H16 wild type (red line), variant v6C6 (blue line) and wild type transformed with plasmids expressing mutated genes (*A0689*$_{v6C6}$, *A1373*$_{v6C6}$, *glpK*$_{v6C6}$ and *A3075*$_{v6C6}$) identified in variants v6C6. Cells were cultivated in MSM with 1% (w/v) glycerol and inoculated to a starting OD_{600} of 0.05.

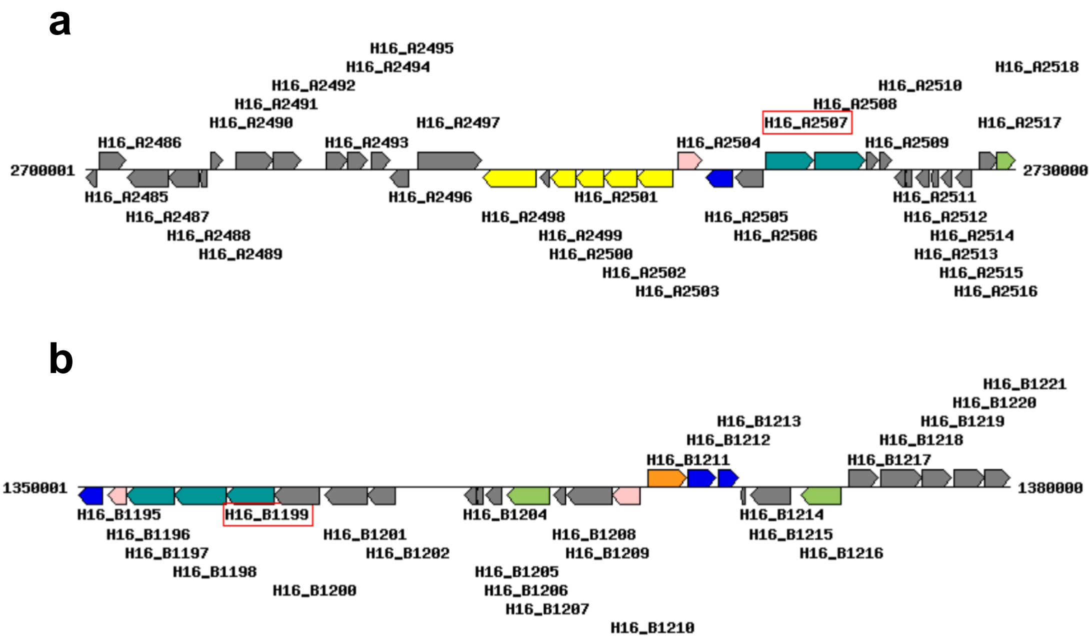

Figure S6: Two pairs of putative glycerol metabolism genes (glycerol kinase and glycerol-3-phosphate dehydrogenase) in *C. necator* H16. The first pair (**a**) has the gene loci of H16_A2507 and H16_A2508 and the second pair (**b**) H16_B1198 and H16_B1199. Genes encoding glycerol kinase are boxed in red.

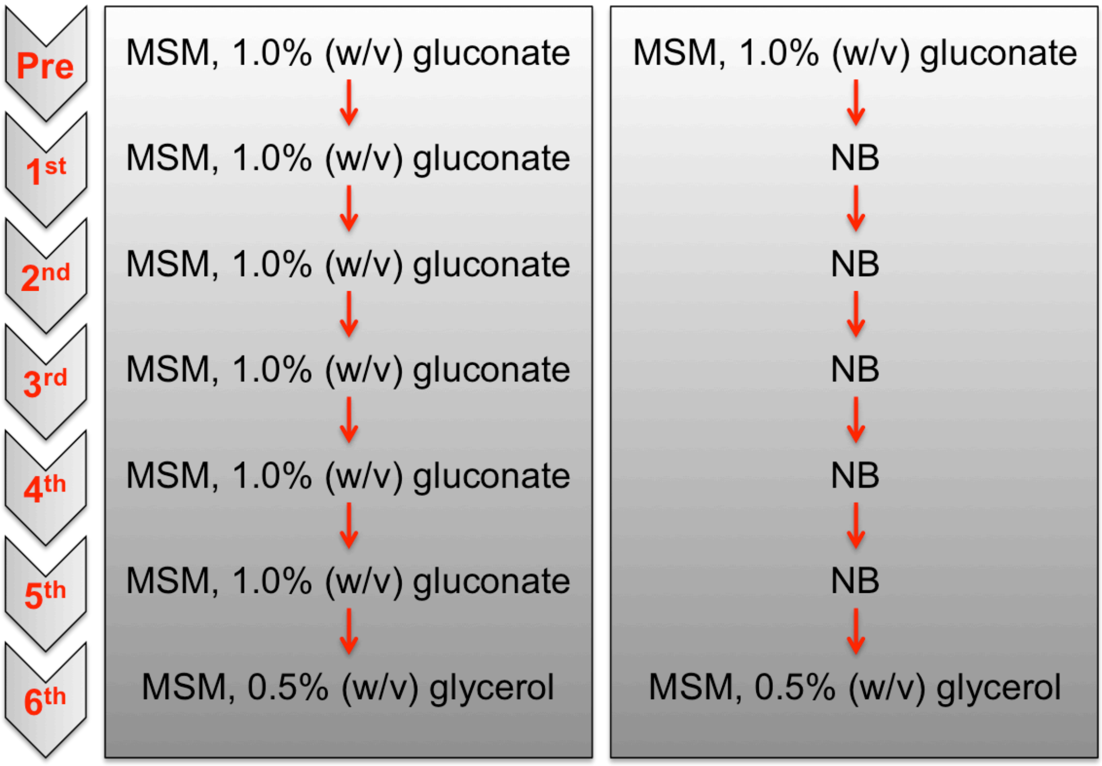

Figure S7: Confirmation of improved glycerol-utilizing phenotype. Cells were grown in synthetic media [1.0 % (w/v) sodium gluconate] or nutrient broth (NB) for 5 rounds of cultivation; on the sixth round, cells were transferred to 0.5 % (w/v) glycerol to confirm stable glycerol-utilizing phenotype of *C. necator* H16.
